# AUPHA and Globalization: A Perspective on the Future

**DOI:** 10.3389/fpubh.2019.00048

**Published:** 2019-04-24

**Authors:** Gerald L. Glandon

**Affiliations:** AUPHA, Washington, DC, United States

**Keywords:** healthcare cost quality and access, global competencies, faculty development, networking activities, idea sharing

## Abstract

The Association of University Programs in Health Administration (AUPHA) celebrated its 70th year in 2018 with a publication of a book on its history (*Looking Back to Look Forward: AUPHA at 70*) (1). As part of that reflection, we engaged in a fundamental reconsideration of our current and future leadership role in supporting healthcare management education. The consideration gets to the core of why any association exists and how does an association best serve the needs of its constituencies. The purpose is to determine what lessons AUPHA's evolution has for healthcare management education associations globally. This perspective will address three questions: 1) What is AUPHA and what is its role in the US healthcare system? 2) What is the value proposition for AUPHA? 3) What lessons learned does AUPHA have to share about the contribution of an association to global health management education?

## What Is AUPHA?

Association of University Programs in Health Administration's (AUPHA) is a free-standing association that represents graduate and undergraduate academic programs that train future healthcare executives. AUPHA has matured over 70 years from an idea originating with a handful of concerned and motivated healthcare leaders in the late 1940s to an organization that has an established reputation and a solid position in the healthcare landscape. It has evolved in terms of its roles and value as a national association of healthcare management experts united by a common mission. AUPHA has a structure of membership, finances and staff to support that mission. It seeks to discover and engage in activities that primarily benefit member programs in implementing education as it pertains to the United States of America (USA) and global settings.

AUPHA's contribution to global health management education is best understood within the context of health management education and practice in the USA. The healthcare system in the USA combines public and private enterprises. Healthcare executives are one of the few, if not the only, health professions not licensed at the federal or state (local) level. Moreover, the training of healthcare executives is neither regulated nor paid for by the government in the US. Formal training of healthcare executives occurs in a variety of educational programs, from undergraduate programs in health administration to graduate programs in many settings, to executive training for clinicians and others seeking leadership roles. Healthcare management students as well as the faculty and the practitioners who mentor them come from a wide range of academic disciplines, including clinical ones. AUPHA is the only nationwide organization that brings together all of those engaged in the education of healthcare executives.

AUPHA membership consists of individual academic programs and individual faculty (most of whom work in the member organizations). AUPHA encompasses over 240 member programs of various types and nearly 2,500 individual faculty. Heterogeneity of membership is one of AUPHA's strengths with programs in schools of health professions (47%), public health (17%), business (18%) and “other” (17.0%). The composition is about 43% female and 76% White. Further, faculty have a variety of degrees (mostly PhD but many MD, nursing, JD, DrPH, EdD, MBA/MHA, and other). Finally, regardless of degree, the faculty disciplines range from finance, accounting, economics, sociology, psychology to human resources, information technology, and supply chain management, to name a few.

## The Role of Healthcare Associations in the USA

The USA healthcare landscape is filled with associations. An association is defined in the federal tax code as an entity that exists to service a constituency rather than make a financial profit. Net revenues are reinvested in the activities of the association. AUPHA concentrates on activities that provide benefit to its members but are difficult for a member to accomplish alone and are not provided by other market participants. The theory suggests associations engage in collective or public goods that competing entities cannot accomplish. As a neutral central party, the association can pool resources and provide services that benefit everyone. Branding the field or lobbying are traditional examples. Although the country has many other health associations provide education to members, AUPHA is the only association that covers education of healthcare executives of all levels, all disciplines, and all specialties.

### Value Proposition for AUPHA

Any organization is defined through its Vision, Mission, and Values. AUPHA continues through its member organizations to develop leaders with the values and competencies necessary to drive improvement throughout the health system. These values and competencies reflect the realization that healthcare involves the public trust. Those responsible for healthcare organizations must lead an efficient organization and thus must have the competencies of any business leader. In addition, they must also have the values to attain and maintain public trust. AUPHA incorporates the *values of excellence, innovation, collaboration, diversity and learning* to build that trust in the educational endeavor (2).

AUPHA's staff and volunteers pursue its mission by generating value to members through a number of key activities: educational and networking events, scholarship, accreditation/certification, scholarships and recognition, creating leadership opportunities, advertising jobs, and special projects. These are highlighted as they pertain primarily within the USA, and the subsequent section discusses how these activities are extended globally.

### Networking

Members benefit from the enhanced ability to share information and learn from one another. This key function constitutes the principle membership benefit and occurs both “virtually” and in person. The Faculty Forums and Network provide a virtual space for faculty, program directors and others to engage in a variety of information sharing, fostering innovative teaching techniques, evolving content and professional contacts while in the comfort of their office or home. The Faculty Forums arose because many individual faculty members have few colleagues in their discipline, host programs, colleges, or universities. The geographic separation of programs and cost of travel to traditional meetings adds to Forum value. The Forum is topic or issue specific and is sanctioned for a specified duration to accomplish this objective. Membership in a Faculty Forum is based exclusively upon faculty electing to participate and the Forums are self-governing. There are currently 15 Faculty Forums.

1. Advancing Women Leaders in Healthcare
2. Cultural Perspectives
3. Ethics
4. Finance, Economics, and Insurance
5. Global Healthcare Management
6. Health Information Management
7. Health Policy
8. Innovative Teaching
9. Interprofessional Education
10. Management
11. Medical Group Practice/Ambulatory Care
12. Online Teaching and Technology
13. Post-acute Care
14. Public Health
15. Quality Improvement

In addition to these formally endorsed Faculty Forums, AUPHA supports an Open Forum for all AUPHA members, as well as communities and discussion groups that seek to establish sufficient faculty engagement for promotion to Faculty Forum status.

Hosting of professional meetings constitute the second major networking activity that AUPHA supports. AUPHA hosts three major events, the Annual Meeting, Graduate Program and Practitioner Workshop, and Undergraduate Workshop, designed for faculty to present teaching techniques, research and other education efforts. They also become intense networking opportunities. The Annual Meeting varies geographically so that all members can reasonably expect to attend a meeting close to home occasionally. Likewise, the Undergraduate Workshop moves to different locations. The Graduate Program and Practitioner Workshop traditionally occurs during the annual Congress on Healthcare Leadership sponsored by the American College of Healthcare Executives (ACHE) in March each year in Chicago.

### Scholarship

AUPHA uses a variety of publications to communicate and disseminate research and other vital health management information. Its primary mechanisms include the *Journal of Health Administration Education (JHAE)* and the *AUPHA Exchange*. Both have migrated from print to electronic editions.

*JHAE*, an on-line journal, is available to all program faculty and individual members as a part of their member benefits. It contains a variety of research and commentary on the state of the art in teaching healthcare management. It also regularly brings in content from the healthcare environment as a means of demonstrating how best to integrate these changes. Healthcare reform, innovation and technology are common topics.

The *AUPHA Exchange* provides a communication vehicle for membership about key issues relevant to the association. Features include program news, association news, and healthcare management employment opportunities. Each monthly edition of the *AUPHA Exchange* contains a blog from the sitting AUPHA Board Chair and the current AUPHA President, news on Board activities, information regarding upcoming AUPHA meetings, network information, a call for nominations if relevant, a new member welcome, and program news.

### Accreditation/Certification

AUPHA began the formal accreditation of graduate healthcare management programs in the 1960s. That function was spun off into a separate organization, Commission on Accreditation of Healthcare Management Education (CAHME). While independent with its own board, CAHME has continued strong ties to AUPHA. It receives substantial financial support from AUPHA each year, and AUPHA members constitute a major portion of its operating committees and board. Similarly, AUPHA has engaged in undergraduate certification since the 1990s, and the process has evolved to become an essential form of recognition. Accreditation and certification have the common goal of improving the quality of healthcare management education. They both assure all external stakeholders that an outside review determined that the program meets or exceeds a set of standards, which assess quality and relevancy. Accreditation and certification further attest that the program withstood the rigors of peer review in which experts critically examine curricula, faculty, and educational outcomes.

### Recognition

An often-overlooked feature of an association is the recognition and celebration of members. AUPHA offers a number of prizes and awards for students and faculty (see Box 1). They are all designed to draw attention to the field, attract potential faculty and potential students. Some of these have long histories while others are relatively recent (For more information and descriptions, see https://www.aupha.org/main/auphanetwork/faculty and https://www.aupha.org/resourcecenter/currentstudents/scholarships).

Box 1 **AUPHA supported prizes and awards for students and faculty.**

William B. Graham Prize for Health Services Research

Andrew Pattullo Lecture

Gary L. Filerman Prize for Educational Leadership

John D. Thompson Prize for Young Investigators

Foster G. McGaw Scholarships

HCA (Hospital Corporation of America) Corris Boyd Scholars Award

Bachrach Family Scholarship for Excellence in Health Administration

David A. Winston Health Policy Scholarships

Upsilon Phi Delta (UPD) Honor Society

### Leadership

AUPHA might be characterized as a member-run organization. A series of standing and special committee empower members to take responsibility for specific tasks. In addition to a Governing Board, AUPHA has basic governance committees such as the Leadership Development, Finance, and a committee for most of its essential activities such as the JHAE Editorial Board. The committees provide opportunities for individual members to develop leadership skills and interact with faculty and practitioners from around the country. Many life-long friendships have been developed among those serving on a committee. Given a relatively small field, the committee structure and shared leadership have built a camaraderie that spans the nation.

### Job Opportunities

AUPHA spreads the word about employment opportunities for faculty, as well as practitioners. Current openings are posted on the website and are noted in the AUPHA Exchange. Members are able to post job openings at their institutions as well as see openings elsewhere. AUPHA is the single best source for announcing and finding employment opportunities in educational institutions.

### Special Projects

Special projects are more targeted and/or more time-limited. This category includes the Body of Knowledge, benchmarking and HAMPCAS (the healthcare administration, management, and policy centralized application service). The application service provides a substantial expansion of the reach and access of students to programs throughout the world. Electronic applications that this system implements facilitate multiple applications by prospective students. Each of these ongoing activities provide direct or indirect support to member programs and faculty.

## AUPHA and Global Associations

Globalization is a priority for AUPHA, as per our most recent strategic plan (3). AUPHA has had a significant global presence for several decades. The founding CEO, Dr. Gary Filerman, was committed to the transfer of healthcare management education thus AUPHA had teams in Eastern Europe and in many of the “newly” independent states from the former Soviet Union. AUPHA also had extensive educational outreach in Latin and South America.

More recently, AUPHA has coalesced global interest by creating the Global Healthcare Management Faculty Forum (GHMFF), one of the 15 forums mentioned earlier. In any given year, AUPHA has an average of 60–70 faculty with experience in providing healthcare management education and/or collaborative research efforts around the world. Each year prior to the AUPHA Annual Meeting, the GHMFF hosts a pre-conference Global Symposium. For a small supplemental fee, this is open to all members with an interest in international pursuits. The attendance at this meeting is small but it has had consistent representation from colleagues in Europe, Latin/South America, Asia and Australia.

In addition to the formal activities sponsored by AUPHA, individual members have come together to further global education. A new textbook, *Global Health Management Education*, will be forthcoming in late 2018, with chapters written by AUPHA members. The intent is to provide a current resource with which to teach healthcare management across national boundaries. This special issue of *Frontiers in Public Health Education and Promotion* on Global Health Management Education emanated from the AUPHA Global Symposium of June 2017, with the chairperson of the GHMFF as one of the co-editors.

Many AUPHA faculty who have worked internationally, have learned that the transfer of knowledge goes in both directions. Faculty return from other countries with ideas and concepts that greatly expand their thinking and influence their teaching. Similarly, the academic programs that offer student placements in other countries consistently find that their students return with sharpened insights into management, cultural sensitivity, collaboration, and governance, among other management subjects.

## AUPHA's Role in Global Healthcare Management Education

The core of AUPHA has been to foster improvement in healthcare management education and thus improvements in healthcare. The strategies of networking, scholarship, accreditation/certification, recognition, leadership building, employment announcements, and special projects that AUPHA currently pursues apply to contexts beyond the USA. Exchanging our expertise with colleagues in other parts of the world is a natural extension of our current activities. Our activities of the past 70 years have resulted in Lessons Learned that we offer to those within the USA as well as colleagues around the world.

- For a profession that is not licensed, formal academic training with rigorous attention to the unique knowledge, skills, and attitudes of the graduates is essential to give the profession credibility. An association represents the profession and the educational institutions with more authority than any university alone. Naturally, the specific aspects of knowledge, skills, and attitudes will differ because of institutional and cultural differences globally.
- Self-governance of adherence to the academic standards set forth for the education becomes imperative when a profession is not licensed by government or other external authorities and can be taught in a variety of university programs by faculty from numerous disciplines. An association can assert “quality control” over education in a way that a single university cannot. Moreover, because licensing is linked to defined governments, the quality standards created by and championed by an association can have more authority on an international level than a license within a single country. AUPHA has gone so far with quality assurance in education to spawn CAHME, but CAHME depends on AUPHA not only for financial support but also for its members, who develop the accreditation criteria, act as site visitors, and keep standards moving as the field changes. In a country that does not follow our particular voluntary accreditation system, an association can still provide the mechanism to influence quality standards.
- In a relatively small field, sharing of information is critical. An association can provide the infrastructure for those in the field to communicate on an ongoing basis and exchange information on everything from model syllabi to recruiting criteria to faculty salaries. The infrastructure provided by AUPHA's Faculty Forums could easily be adapted to participation by faculty in other countries.
- In a profession where the members are often uncounted, an association can provide an institutional rallying point, drawing members who are committed to management practice rather than a particular clinical or academic discipline.
- Collaboration rather than Competition. Those who work in healthcare tend to be driven by values and a commitment to fellow man. Personalities and education tend to be different for those in healthcare than for those in commercial enterprises. Collaboration tends to override competition. An association can foster and reinforce collaboration as the social norm of the profession.

## Conclusions

By bringing together professionals from a variety of professional and academic settings and with a wide array of disciplines, our open framework empowers all participants to “assemble” and share their individual experiences. Meeting face-to-face and/or participating in the virtual network provides an opportunity for faculty from all countries to brainstorm about content, exchange syllabi, and struggle to define the competencies necessary for future healthcare leadership. AUPHA has a long history and a well-developed format for guiding healthcare management education in the USA.

Our educational model is hardly perfect, however. The challenges that the US healthcare system faces today resulted from or were enabled by leaders trained largely by our programs over the last 70 years. Many others share major portions of the responsibility for these challenges it should be added. More informed and more socially conscious leadership in past decades might have mitigated portions of the cost, quality and access problems we currently confront.

It is clear that managing the complex healthcare systems throughout the world presents different but strongly related challenges in every country. As such, healthcare leadership globally can benefit from the experiences of those leading our system. Similarly, our constantly improving healthcare management educational systems can serve as a model for those training healthcare leadership in other countries. AUPHA and its members currently share their expertise worldwide and are eager to expand that sharing. All would benefit from commentary, sharing of content and camaraderie provided by connecting with healthcare management faculty and practitioners throughout the world. AUPHA faculty are keenly aware that the benefits are not in one direction. We, too, can learn, obtain innovative perspectives and become more effective by collaborating with educators around the world. Given healthcare outcomes reported from many sources, many countries provide better access to care, equal or better quality outcomes at much lower costs than the US. We are excited about identifying the best features of our very different systems to the benefit of the health of the world's population.

## Author Contributions

The author confirms being the sole contributor of this work and has approved it for publication.

### Conflict of Interest Statement

GG is President and CEO of AUPHA, the subject of the perspective.
